# Hyperhomocysteinemia and Vitamin B Deficiency as Potential Aggravating Factors in Huntington's Disease: A Prospective Monocentric Study

**DOI:** 10.1002/mds.70413

**Published:** 2026-07-01

**Authors:** Salomé Puisieux, Elise Pourié, Céline Bonnet, Lucie Hopes, Solène Frismand, Myriam Bronner, Virginie Roth, Marion Wandzel, Natacha Sloboda, Laetitia Lambert, Abderrahim Oussalah, Amélia Julien, Irina Rotaru, Rosa‐Maria Guéant‐Rodriguez, Jean Louis Guéant, Carine Bossenmeyer‐Pourié, Mathilde Renaud

**Affiliations:** ^1^ Department of Neurology University Hospital of Nancy Nancy France; ^2^ INSERM UMR_S 1256, Nutrition, Genetics, and Environmental Risk Exposure (NGERE), Faculty of Medicine of Nancy University of Lorraine Nancy France; ^3^ Laboratory Genetics University Hospital of Nancy Nancy France; ^4^ Department of Clinical Neurogenetics Hospital of Metz F‐57530 Ars‐Laquenexy France; ^5^ Department of Clinical Neurogenetics University Hospital of Nancy Nancy France; ^6^ Medical Laboratory Reference Center for Inborn Errors of Metabolism (ORPHA67872), University Hospital of Nancy Nancy France; ^7^ Department of Genomic Medicine, Division of Biochemistry, Molecular Biology, and Nutrition University Hospital of Nancy Nancy France; ^8^ Tecnologico de Monterrey Institute for Obesity Research Monterrey Mexico; ^9^ Joan Klein Jacobs Center for Precision Nutrition and Health Cornell University Ithaca New York USA

**Keywords:** aggravating factors, homocysteine, Huntington's disease, hyperhomocysteinemia, one‐carbon metabolism, vitamin B deficiency

## Abstract

**Background:**

Although not confirmed, some studies have suggested that elevated homocysteine levels are common in patients with Huntington's disease (HD). Its clinical relevance remains unclear.

**Objectives:**

We aimed to assess vitamin B and homocysteine levels in HD patients and explore the relationships among hyperhomocysteinemia, vitamin B deficiency, and motor symptom progression.

**Methods:**

In this monocentric case–control study, 77 HD patients were prospectively included between January 2020 and June 2023 at Nancy University Hospital. As controls, biological data were obtained retrospectively from 87 individuals referred to the Metabolic Medicine Department between January 2017 and February 2025. HD patients underwent clinical (Unified Huntington Disease Rating Scale‐Motor) and biochemical (vitamin B and homocysteine levels) evaluations. Fisher's exact test, Wilcoxon test, or Student's t‐test were used, with *P* < 0.05 considered significant.

**Results:**

HD patients—both symptomatic and premanifest—had significantly higher homocysteine levels than did controls. Hyperhomocysteinemia was found in 36 patients (≈48%) and was associated with faster motor progression (*P < 0.001*).

**Conclusions:**

Hyperhomocysteinemia appears to be frequent in HD patients and may represent a risk factor contributing to phenotypic variability. These findings support a potential link between altered one‐carbon metabolism and HD. Further longitudinal and mechanistic studies are needed to clarify the clinical and pathophysiological relevance of homocysteine metabolism in HD and to determine whether targeting hyperhomocysteinemia could have therapeutic implications. © 2026 The Author(s). *Movement Disorders* published by Wiley Periodicals LLC on behalf of International Parkinson and Movement Disorder Society.

Huntington's disease (HD) is an autosomal‐dominant neurodegenerative disorder marked by chorea, cognitive decline, and behavioral changes,^1^ caused by a toxic gain‐of‐function mutation in the huntingtin (HTT) protein due to expanded CAG repeats (>39).^2^ Despite its single‐gene origin, HD shows high phenotypic variability and unclear pathophysiology.^3^

Hyperhomocysteinemia (HHcy), linked to disrupted one‐carbon metabolism (1‐CM), is associated with neurodegenerative diseases such as Alzheimer's 4, 5 and Parkinson's disease (PD),^5^, ^6^, ^7^, ^8^ but its role in HD remains underexplored. Homocysteine (Hcy) arises from methionine demethylation in 1‐CM, which includes the folate cycle, methionine cycle, and transsulfuration pathway^9^ (Fig. S1). Methylene‐tetrahydrofolate reductase (MTHFR) converts folates to 5‐methyltetrahydrofolate (5‐MTHF), which remethylates Hcy to methionine via vitamin B12‐dependent methionine synthase (MS), or Hcy enters the transsulfuration pathway, where vitamin B6‐dependent cystathionine β‐synthase (CBS) and cystathionine γ‐lyase (CSE) generate cysteine and glutathione. HTT regulates this pathway^10^ through interactions with CBS and specificity protein 1 (SP1), which controls CSE expression. CSE expression is also controlled by activating transcription factor 4 (ATF4). Under oxidative stress, cysteine is used for glutathione production, triggering ATF4 to upregulate CSE and restore cysteine/glutathione levels.

Any factor impairing 1‐CM—including vitamin B6/B12/folate deficiency, sex, medications, kidney dysfunction, or genetic variants—can elevate the Hcy plasma level (t‐Hcy).^11^

In HD, mutant HTT (mHTT) disrupts the transsulfuration pathway by sequestering SP1 and reducing CSE expression, raising Hcy and reactive oxygen species (ROS) levels.^10^, ^12^ Progressive ROS accumulation also suppresses ATF4.^10^ Altered CBS–mHTT interactions may further impair this pathway.^13^, ^14^ Brune et al.^15^ reported that HD patients with the homozygous *MTHFR‐1298CC* polymorphism have earlier symptom onset.

Considering these data, we hypothesize that nutritional or genetic causes of HHcy may increase HD onset risk and severity. This monocentric case–control study aimed to (1) measure t‐Hcy in symptomatic and premanifest HD patients and (2) examine associations between vitamin B deficiency, HHcy, and motor progression.

## Methods

### Study Design and Subjects

We conducted a monocentric case–control study in which data from HD patients were collected prospectively between January 2020 and June 2023, whereas control data were retrospectively retrieved from medical records between January 2017 and February 2025.

As patients, inclusion criteria were age >18 years, molecularly confirmed HD (CAG ≥36), and follow‐up at our HD Center in Nancy, France. Seventy‐seven *HTT* mutation carriers were enrolled. Exclusion criteria were age <18 years, inability to follow up, pregnancy, or cognitive impairment affecting study comprehension. Patients were assessed during routine follow‐up, with a single expert visit involving clinical data collection, standardized scales, and biological testing, all performed by a certified Unified Huntington's Disease Rating Scale (UHDRS) examiner. Medical chart data were also used.

As controls, biological data from 87 individuals were obtained from Nancy Biochemical Database. Controls were adults (>18 years) referred to the Metabolic Medicine Department for suspected familial dyslipidemia or moderate dyslipidemia associated with statin intolerance. Patients with liver disease, diabetes mellitus, heart failure, heart attack, myopathy, parkinsonism, or ongoing vitamin supplementation at the time of sampling were excluded.

### Ethics

All participants or legal representatives signed non‐opposition forms authorizing the use of data for research purposes. Biochemical data were retrieved from the Nancy Biochemical Database, with ethical approval and Commission Nationale de l'informatique et des libertés (National Commission on Informatics and Liberty) CNIL registration (No. 1763197v0). The study was conducted in accordance with the Declaration of Helsinki.

### Data Collection

Collected data from the HD population included: (1) General clinical data (patient's medical history, family medical history, current treatments, previous treatments, patient/relative‐reported date of first symptoms, patient/relative‐reported date of motor symptoms, smoking, consumption of coffee, coke, and energy drink. (2) First symptom(s) reported by patients/relatives: (a) Motor symptoms corresponding to at least one of the following: chorea, dystonia, rigidity, akinesia, gait disorders. (b) Cognitive dysexecutive syndrome corresponding to at least one of the following symptoms: psychomotor slowing, attentional disorders, impairment of working memory, impairment of mental flexibility and mental strategy, or mental rigidity. (c) Behavioral dysexecutive syndrome corresponding to at least one of the following symptoms: impulsivity, irritability, aggressiveness, social behavior disorders, emotional lability, and apathy. (d) Anxious and/or depressive symptoms. (3) The Unified Huntington Disease Rating Scale‐Total Motor Score (UHDRS‐TMS),^16^ which was used to determine the severity of motor symptoms in HD patients. The UHDRS‐TMS provides a uniform clinical rating of the motor function of HD patients. It includes 15 items to assess symptoms such as chorea, dystonia, and rigidity, and each item is rated on a scale ranging from 0 (“normal”) to 4 (“cannot perform/severe problems”). Higher scores indicate increased motor dysfunction. We defined premanifest patients as patients with UHDRS‐TMS ≤ 5^17^ and symptomatic patients as patients with UHDRS‐TMS > 5. (4) Cytobiochemical data (complete blood count, C‐reactive protein, albumin, proalbumin, t‐Hcy, methylmalonic acid [MMA], vitamin B12, vitamin B6, red blood cell (RBC) folate and serum folate levels, estimated glomerular filtration rate, and liver enzymes [ASAT and ALAT], lipid profile).

Collected data from the control population included: demographic characteristics (age and sex), serum folate, vitamin B12, MMA levels, t‐Hcy, estimated glomerular filtration rate, and liver enzymes (aspartate aminotransferase [ASAT] and alanine aminotransferase [ALAT]).

All laboratory analyses (control and cases) were conducted in a single laboratory, with no modifications to the measurement techniques throughout the study period.

To compare motor progression between patients, we estimated each patient's motor progression rate corresponding to the UHDRS‐TMS at inclusion divided by the duration of motor symptoms (UHDRS‐TMS/MD). The duration of motor symptoms was estimated on the basis of the declarative onset of motor symptoms collected from the patient and/or relatives. Because the UHDRS‐TMS loses linearity beyond the early stages of HD^18^, ^19^, ^20^, ^21^, ^22^ due to ceiling effects, we restricted our analysis to patients with less than 5 years of motor progression. During this period, longitudinal studies consistently show an approximately linear increase in TMS, supporting the use of annualized progression rates. Previous works^23^, ^24^, ^25^ confirmed that TMS changes are reliably linear and valid markers of disease progression in early‐stage HD. Furthermore, restricting our analysis to HD patients with a disease duration of less than 5 years minimized recall error in onset dating.

### Laboratory Assays

Vitamin B12/folate was measured via Immunoassay Reagent Atellica® (Siemens), B6 by high‐performance liquid chromatography (HPLC) (Shimadzu), and Hcy and MMA by ultra‐performance liquid chromatography (UPLC)‐MS/MS via an ACQUITY UPLC BEH C18 column (1.7 μm, 2.1 × 50 mm; Waters Corporation). HHcy was defined as t‐Hcy >15 μmol/L, B6 deficiency <20 nmol/L, RBC folate <270 nmol/L, B12 deficiency <150 pmol/L. Other laboratory tests included C‐reactive protein (CRP), albumin, glomerular filtration rate (GFR) via the Chronic Kidney Disease–EPIdemiology formula (CKD‐EPI), hemoglobin, and MCV (Atellica Analyser, Siemens).

### Statistical Analyses

Categorical variables are reported as counts (%), and continuous variables as mean ± standard deviation (SD). Differences between controls and HD patients, and between HD patients with or without HHcy or vitamin B deficiency, were assessed using Fisher's exact test for categorical and Wilcoxon or Student's tests for continuous variables.

To assess the rate of motor progression over time (UHDRS‐TMS/MD) across the HD patients according to Hcy and vitamin B status, four groups were defined: (1) Hcy and vitamin B normal; (2) isolated HHcy; (3) vitamin B deficiency without HHcy; and (4) HHcy and vitamin B deficiency. To statistically compare progression between groups, linear regression models (adjusted for age/sex) were performed with the score as the dependent variable and group as the independent variable, using Group 1 as the reference. Model assumptions were checked, and robust standard errors were applied when appropriate.

We performed multivariate analysis using Spearman's rank correlation test to assess the relationships among the clinical, genetic, and biochemical data (global n = 77, symptomatic n = 62, premanifest n = 15). Additionally, multiple regression analyses were performed in the overall study population to assess whether age, sex, tobacco consumption, coffee intake, and coke consumption were independently associated with plasma homocysteine levels. In symptomatic patients, additional analyses were conducted to investigate the associations between UHDRS‐TMS/MD scores and CAG repeat length, plasma homocysteine, folate, vitamin B6, and vitamin B12 levels. To account for multiple comparisons, we applied the Benjamini–Hochberg false discovery rate. Two‐sided *P* < 0.05 was considered significant. Analyses were done in XLSTAT and Stata (version 19.5) (StataCorp, College Station, TX, USA).

## Results

### Clinical and Paraclinical Features of *HTT* Gene Mutation Carriers

Seventy‐seven *HTT* mutation carriers were included (Table 1). Fifteen carriers were premanifest (UHDRS‐TMS < 5). A family history of HD was reported in 85% of participants (65/77), with maternal transmission in 47% (36/77) and paternal transmission in 38% (29/77). Fifty‐eight patients drank coffee every day (2.3 ± 2.4 cups/day), 7 patients also drank coke (4.9 ± 2.6 glasses/day), and only 1 patient drank energy drinks (3 glasses/day). Eighteen patients smoked, with an average of 12.9 ± 6 cigarettes per day. Among the 62 symptomatic patients, the mean age at onset was 43.9 ± 13.6 years, with a mean UHDRS‐TMS of 26.7 ± 20.6. HHcy was present in 39/77 patients (51%), with a mean Hcy level of 18.2 ± 20.6 μmol/L. Multivariate analysis revealed a moderate negative correlation between CAG allele 1 repeats, whereas age and CAG allele 2 were not statistically significant and t‐Hcy (r = −0.311; *P < 0.05*); and a strong negative correlation with age of onset (r = −0.727; *P < 0.001*). At the time of biological sampling, none of the patients were receiving vitamin B supplementation.

**TABLE 1:** Characteristics of the Huntington's disease patients (N=77) included in the study

Demographic data	n/N	Value
Age (years), mean ± SD		50.7 ± 13.7
Sex (M/F), n		31/46
Age at onset of HD (years), mean ± SD	71/77	43.9 ± 13.5
Age at onset of motor symptoms (years), mean ± SD	62/77	46.9 ± 12.6
Family history of HD, % (n)	77/77	85 (65)
Maternal transmission	77/77	47 (36)
Paternal transmission	77/77	38 (29)
CAG repeat, longer allele, mean ± SD	77/77	43.5 ± 4.1
CAG repeat, shorter allele, mean ± SD	66/77	18.9 ± 4.0
HD duration (years), mean ± SD	71/77	6.5 ± 5.7
UHDRS‐TMS, mean ± SD	77/77	26.7 ± 20.6
Smoker, % (n)	77/77	23 (18)
Cigarettes per day, mean ± SD	18/77	12.9 ± 6.0
Coffee drinker, % (n)	77/77	75 (58)
Coffee (cups per day), mean ± SD	58/77	2.31 ± 2.37
Coke drinker, % (n)	77/77	9 (7)
Coke (glasses per day), mean ± SD	7/77	4.9 ± 2.6
Energy drink consumer	77/77	1,3 (1)
Energy drink (glasses per day), mean ± SD	1/77	3
Current treatments affecting homocysteine/vitamin B metabolism, % (n)	77/77	
Methotrexate	77/77	0
Phenytoin	77/77	0
Carbamazepine	77/77	0
Phenobarbital	77/77	0
Metformin	77/77	0
Fibrates	77/77	0
Hydrochlorothiazide	77/77	0
Levodopa	77/77	1.3 (1)
Vitamin B6 level, >20 nmol/L, mean ± SD	75/77	36 ± 29.4
RBC folate level, >270 nmol/L, mean ± SD	77/77	918 ± 262
Folates, serum level, >7 nmol/L, mean ± SD	63/77	19.8 ± 10.3
Vitamin B12 level, >150 pmol/L, mean ± SD	77/77	340 ± 137
Homocysteine level, <15 μmol/L, mean ± SD	77/77	18.2 ± 20.6
Methylmalonic acid, μmol/L, mean ± SD	76/77	0.36 ± 1.24
Anormal methylmalonic acid, % (n)		1.3 (1)
Hemoglobin, g/dL, mean ± SD	76/77	13.5 ± 3.3
MCV, fL, mean ± SD	76/77	89.8 ± 7.5
Macrocytosis, % (n)		3.9 (3)
Microcytosis, % (n)		3.9 (3)
GFR, mL/min, mean ± SD	57/77	87.7 ± 6.8
No renal failure, >89 mL/min, % (n)		77.2 (44)
Mild renal failure, 60–89 mL/min, % (n)		19.3 (11)
Moderate renal failure, 30–59 mL/min, % (n)		3.5 (2)
Albumin, g/L, mean ± SD	61/77	41.9 ± 4.4
Prealbumin, g/L, mean ± SD	73/77	0.27 ± 0.06
C‐reactive protein, mg/L, mean ± SD	71/77	4.9 ± 8.15
ASAT, U/L, mean ± SD	51/77	22.1 ± 8.8
ALAT, U/L, mean ± SD	51/77	23.2 ± 14.7
Total cholesterol, g/L, mean ± SD	42/77	1.94 ± 0.59
HDL cholesterol, g/L, mean ± SD	42/77	0.53 ± 0.13
LDL cholesterol, g/L, mean ± SD	42/77	1.22 ± 0.45
Triglycerides, g/L, mean ± SD	42/77	1.29 ± 0.87
Biological abnormalities, % (n)		
Isolated hyperhomocysteinemia		29.9 (23)
Isolated vitamin B6 deficiency		9.1 (7)
Isolated vitamin B9 deficiency		0
Isolated vitamin B12 deficiency		2.6 (2)
HHcy and vitamin B12 deficiency		1.3 (1)
HHcy and vitamin B6 deficiency		16.9 (13)
HHcy and vitamin B12 and B6 deficiencies		1.3 (1)
HHcy and vitamin B12 and B9 deficiencies		1.3 (1)

Abbreviations: SD, standard deviation; M, male; F, female; HD, Huntington's disease; UHDRS‐TMS; Unified Huntington Disease Rating Scale‐Total Motor Score; RBC, red blood cell; MCV, mean cell volume; GFR, glomerular filtration rate; ASAT, aspartate aminotransferase; ALAT, alanine aminotransferase; HDL, high‐density lipoprotein; LDL, low‐density lipoprotein; HHcy, hyperhomocysteinemia.

### Clinical and Biochemical Features of Control Population

Eighty‐nine controls were included in the study (Table S1). The mean age was 50.2 ± 12.2 years, and 58.6% were male (51/87). Mean t‐Hcy was 12.8 ± 2.7 μmol/L, while mean vitamin B12 and serum folate levels were 338.8 ± 95.9 pmol/L and 20.8 ± 7.4 nmol/L, respectively. Isolated hyperhomocysteinemia was observed in 17.2% of controls (15/87), whereas no isolated vitamin B9 or vitamin B12 deficiencies were identified. Mean MMA level was 0.16 ± 0.06 μmol/L, with no abnormal values detected. Renal function was preserved overall, with a mean GFR of 92 ± 7.4 mL/min; mild renal failure (60–89 mL/min) was present in 11.9% of controls (8/67), while no renal failure (30–59 mL/min) was observed.

### Clinical, Nutritional, Metabolic, and Renal Causes of HHcy

Among 77 HD patients, 23 (29.9%) had isolated HHcy and 16 (20.8%) HHcy with vitamin B deficiency: 13 (16.9%) with B6 deficiency and one each (1.3%) with B12, folate+B12, and B6 + B12 deficiencies. Isolated vitamin B deficiency occurred in 9 patients (11.7%). No B12 deficiency was associated with anemia or elevated MMA; one patient showed isolated MMA elevation, and none were anemic. Renal function (n = 57) was normal in 77.2% (n = 44), mildly impaired in 19.3% (n = 11), and moderately impaired in 3.5% (n = 2), with HHcy present in only one patient with moderate impairment (t‐Hcy 18.4 μmol/L). Only one patient was receiving a treatment known to affect homocysteine and vitamin B levels. This patient, who was treated with levodopa, presented with HHcy (31.7 μmol/L) without evidence of vitamin B deficiency and carried a pathogenic CBS variant (c.833 T>C, p.Ile278Thr, Class 5). In the multiple logistic regression analysis, none of the investigated factors (age, sex, tobacco consumption, coffee intake, coke consumption) showed a significant independent association with HHcy.

### Comparison between HD Population and Controls

Mean t‐Hcy was significantly higher in HD patients than in controls across the whole cohort (16.6 ± 8.3 vs. 12.8 ± 3.6 μmol/L; *P* < 0.01; Table 2 and Fig. 1). This difference remained significant when symptomatic HD patients and premanifest HD patients were analyzed separately (both *P* < 0.05). In contrast, serum vitamin B12 and folate levels did not differ significantly between HD patients and controls. HHcy prevalence was greater in HD men (51.6%, 16/31) and HD women (50%, 23/46) than in control men (17.6%, 9/51) and control women (19.4%, 7/36) (all *P* < 0.01). In multiple regression adjusted for age and sex, HD status remained significantly associated with higher homocysteine levels. Linear regression models with log‐transformed biomarkers and robust standard errors were used.

**TABLE 2:** Comparison between Huntington's disease population and controls

	HD population (n = 77)			Control population (n = 87)			
Parameter	n	Median	Mean ± SD	n	Median	Mean ± SD	*P‐*value
Sex (M/F)	77	–	46 M/31F	87	–	51 M/38F	0.838
Age (years)	77	52.0	50.6 ± 13.7	87	51.3	50.0 ± 15.4	0.966
Homocysteine level, μmol/L							
All population	77	14.0	16.6 ± 8.3	87	12.3	12.8 ± 3.6	<0.01**
Symptomatic population	62	13.8	16.1 ± 8	87	12.3	12.8 ± 3.6	<0.05*
Premanifest population	15	16.9	18.5 ± 9.4	87	12.3	12.8 ± 3.6	<0.05*
Vitamin B12 level, >150 pmol/L	77	321	340 ± 137	87	307.0	338.2 ± 236.9	0.630
Folate serum level, >7 nmol/L	63	17	19.5 ± 10.2	85	18.0	20.6 ± 10.0	0.376

*Note*: Mann–Whitney U test (MW, two‐sided nonparametric) for continuous variables; *χ* ^2^ test for sex.

Abbreviations: HD, Huntington's disease; SD, standard deviation; M, male; F, female.

* *P* < 0.05,

** *P* < 0.01,

*** *P* < 0.001.

**FIG. 1:**
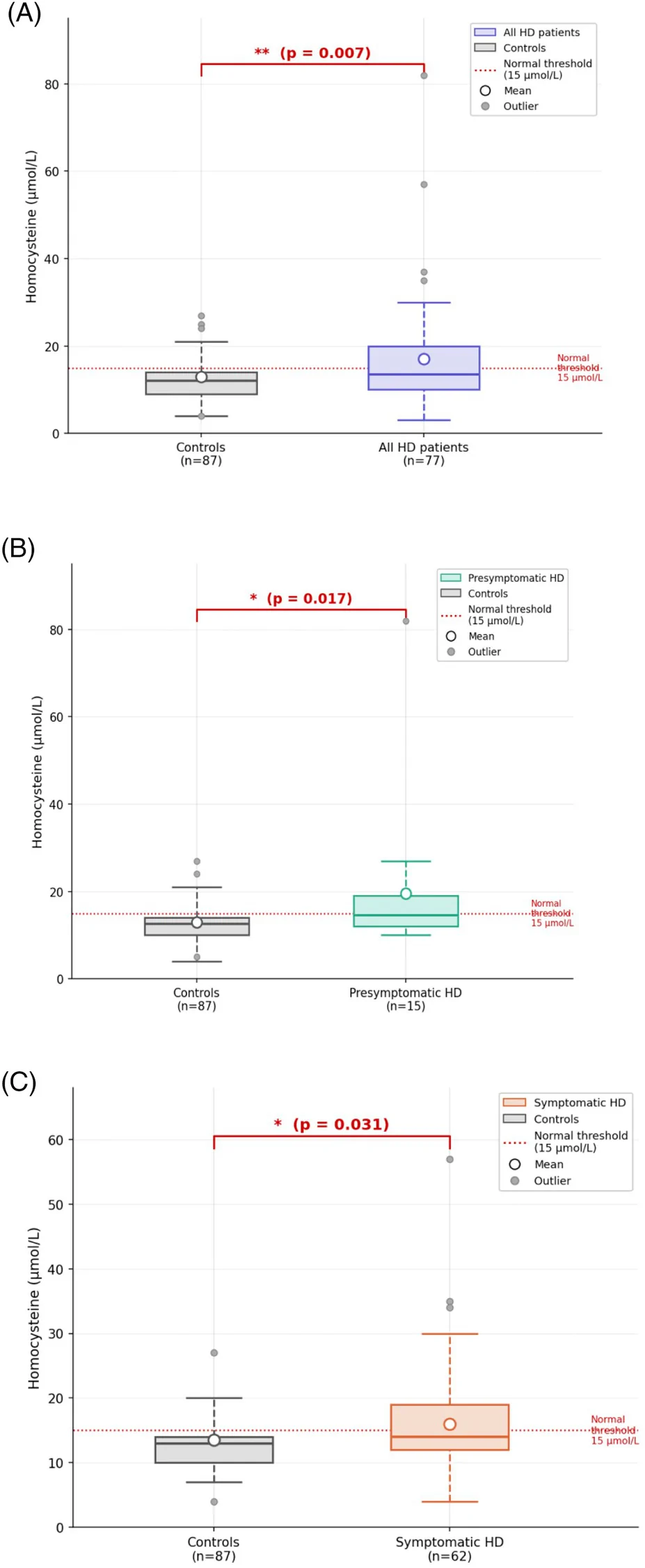
Plasma homocysteine concentrations in Huntington's disease (HD) patients and controls. (A) Boxplots display the median (horizontal line), interquartile range (IQR, box), 1.5 × IQR whiskers, individual outliers (open circles) and group mean (white circle marker). The dashed red line indicates the upper limit of the normal reference range (15 μmol/L). HD patients were stratified as symptomatic (n = 62) or presymptomatic (n = 15). (A) All HD patients (n = 77) versus controls (n = 87); Mann–Whitney U = 4162, *P* = 0.007 (**). (B) Presymptomatic HD (n = 15) versus controls (n = 87); Mann–Whitney U = 906, *P* = 0.017 (*). (C) Symptomatic HD (n = 62) versus controls (n = 87); Mann–Whitney U = 3256, *P* = 0.031 (*). Significance thresholds: *P < 0.05, **P < 0.01, ***P < 0.001. [Color figure can be viewed at wileyonlinelibrary.com]

### Association of HHcy with Clinical and Paraclinical Features in Symptomatic HD

Among 62 symptomatic patients, 30 (48.8%) had HHcy (Table S2), including 15 (24.2%) isolated HHcy. HHcy was predominantly observed in patients with disease duration <5 years (n = 39), accounting for 83% (25/30) of all HHcy and 73% (11/15) of isolated HHcy cases; in this subgroup, HHcy and isolated HHcy prevalences were 64% (25/39) and 28% (11/39), respectively. Beyond 5 years, prevalences declined to 22% and 17%. CAG repeat length did not differ between groups, but patients with HHcy and/or vitamin B deficiency showed significantly faster motor progression (higher UHDRS‐TMS/MD; *P < 0.001*; Table 3). Logistic regression confirmed a strong association between HHcy and high motor progression rate (odds ratio [OR] = 13.66, 95% confidence interval [95% CI] 2.82–66.17; *P = 0.001*), despite the limited sample size of the study population, which was reflected by the large 95% CI.

**TABLE 3:** Characteristics of the symptomatic Huntington's disease population

Characteristic	Patients without HHcy or vitamin B deficiency (n = 23)	Patients with HHcy or vitamin B deficiency (n = 39)	*P‐*value
Age at onset of HD (years), mean ± SD	44.1 ± 9.4	45.1 ± 14.5	0.68
Age at onset of motor symptoms (years), mean ± SD	45.3 ± 12.1	48.2 ± 12.9	0.38
CAG repeat, longer allele, mean ± SD	44.8 ± 2.11	43.8 ± 3.4	0.11
CAG repeat, shorter allele, mean ± SD	18.2 ± 1.67	19.4 ± 5.2	0.66
HD duration (years), mean ± SD	10.1 ± 4.6	6.2 ± 5	<0.05*
Motor symptoms duration (years), mean ± SD	8.6 ± 5.1	3.2 ± 3.5	<0.05*
Vitamin B6, nmol/L, mean ± SD	46 ± 32.6	25.7 ± 16.4	<0.001*
RBC folate level, nmol/L, mean ± SD	1104 ± 264	791 ± 293	<0.001*
Vitamin B12, pmol/L, mean ± SD	416 ± 164	284 ± 135	<0.001*
Homocysteine, μmol/L, mean ± SD	10.5 ± 2.7	19.9 ± 9.3	< 1e−5*
Methylmalonic acid, μmol/L, mean ± SD	0.18 ± 0.1	0.25 ± 0.2	0.36
UHDRS‐TMS, mean ± SD	39.9 ± 18.8	30.7 ± 15.8	0.32
Rate of motor progression, ratio UHDRS‐TMS/motor symptoms duration, mean ± SD	0.41 ± 0.19	1.18 ± 0.95	<0.001*
First reported symptom			
Motor symptoms, % (n)	69.6 (16)	41 (16)	<0.05*
Cognitive dysexecutive syndrome, % (n)	4.3 (1)	5.1 (2)	0.84
Behavioral dysexecutive syndrome, % (n)	8.7 (2)	35.9 (14)	<0.05*
Anxious and depressive symptoms, % (n)	17.4 (4)	17.9 (7)	1

Abbreviations: HHcy, hyperhomocysteinemia; HD, Huntington's disease; SD, standard deviation; RBC, red blood cell; UHDRS‐TMS, Unified Huntington's Disease Rating Scale‐Total Motor Score.

* Considered significant.

In early‐stage HD (<5 years; n = 39), patients were divided into four groups: isolated HHcy, HHcy with vitamin B deficiency, isolated vitamin B deficiency, and those without HHcy or vitamin B deficiency. Isolated HHcy (n = 8) or HHcy with vitamin B deficiency (n = 11) were associated with significantly faster motor progression compared with patients without HHcy or vitamin B deficiency (*P < 0.001*), whereas isolated vitamin B deficiency (n = 5) was not (*P = 0.1*).

HHcy or vitamin B deficiency were also associated with behavioral‐dysexecutive onset (*P = 0.031*) (Table 3).

In multivariate analysis, homocysteine levels correlated moderately with motor symptom progression (r = 0.420; *P < 0.001*) (Fig. 2A), whereas vitamin B levels and CAG repeat length (longer allele) were not significantly correlated (Fig. 2B–E).

**FIG. 2:**
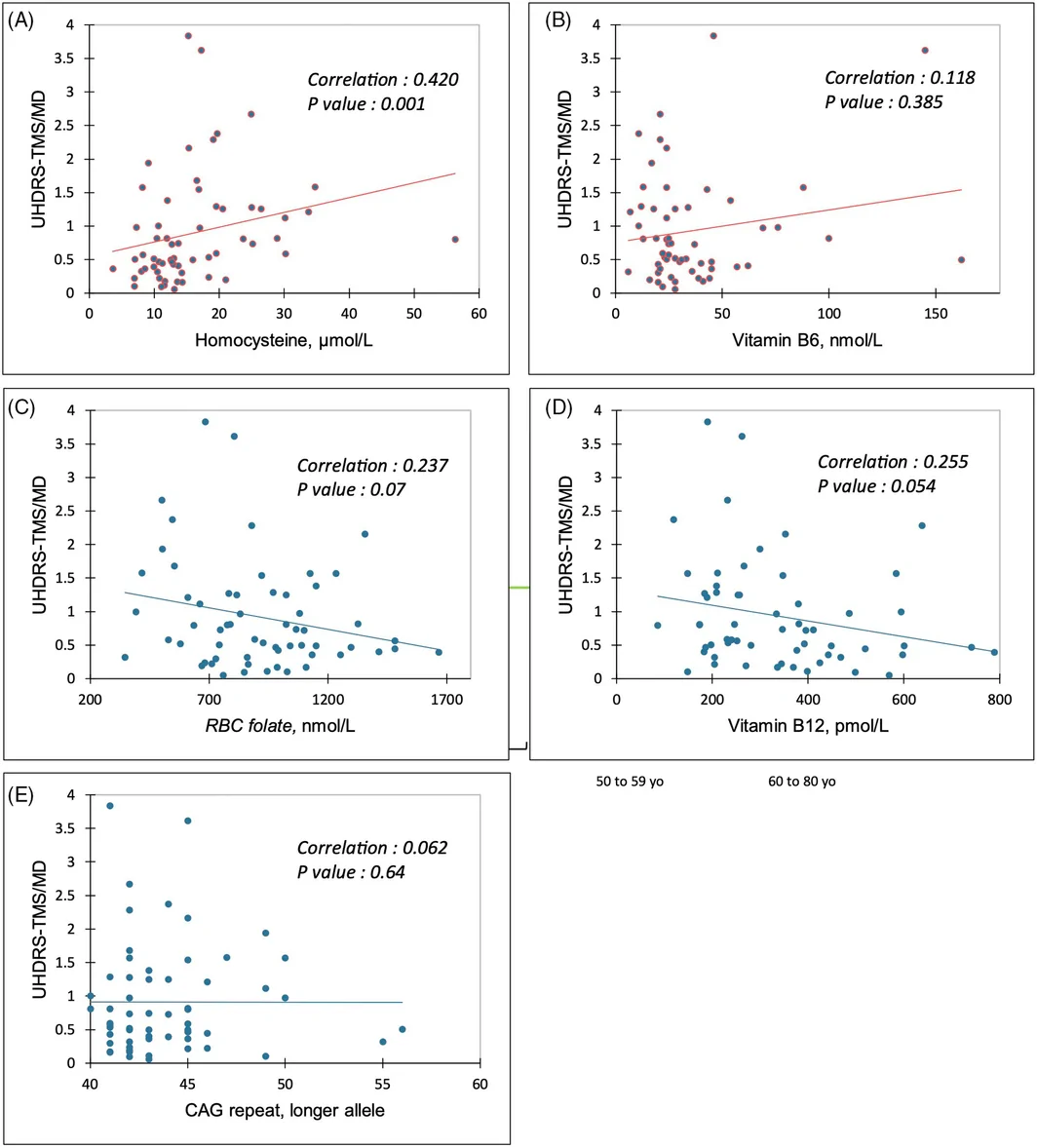
Scatterplots demonstrating the correlation between motor symptom progression (ratio) according to hyperhomocysteinemia and vitamin B deficiency via Spearman's rank correlation coefficient. An n value <0.05 was considered significant. Progression of motor symptoms estimated for each patient, corresponding to the ratio of the Unified Huntington's Disease Rating Scale‐Total Motor Score to the duration of motor symptoms in months (UHDRS‐TMS/MD). Scatterplot showing the relationships between (A) homocysteine levels, (B) vitamin B6 levels, (C) red blood cell (RBC) folate levels, (D) vitamin B12 levels, and (E) CAG repeats (longer allele), and motor symptom progression. [Color figure can be viewed at wileyonlinelibrary.com]

### Association of HHcy with Premanifest HD

Among 15 premanifest patients, CAG repeat length did not differ by HHcy or vitamin B status (Table S3). HHcy was present in 9/15 patients (60%), including 8/15 (53.3%) isolated HHcy—constituting a higher prevalence than in symptomatic patients, though not statistically significant (*P = 0.57*) (Table S4).

## Discussion

This prospective study of 77 patients is the first to examine the associations between HHcy, vitamin B deficiency, and HD onset and progression. We show that t‐Hcy is significantly higher in both symptomatic and premanifest HD patients than in controls across all ages, despite higher B12 and folate levels, and independently of CAG repeat length. Elevated t‐Hcy appears to be a frequent and previously underrecognized feature of HD.

These findings partially contrast with Andrich et al.,^26^ who reported elevated t‐Hcy only in advanced, treated HD patients (n = 34). However, treatment exposure was insufficiently characterized, with only broad therapeutic categories reported (antidepressants, neuroleptics, benzodiazepines, tetrabenazine, and anticholinergics), limiting the interpretation of potential treatment‐related effects on Hcy metabolism. In addition, the absence of elevated t‐Hcy in mildly affected patients may be explained by limited statistical power and inadequate adjustment for major determinants of HHcy, particularly age and sex. Indeed, mildly affected patients in their cohort were younger (44 ± 12 vs. 55 ± 13 years) and included proportionally fewer men (M/F ratio: 8/11 vs. 40/34), both factors known to influence homocysteine levels.

At the cellular level, increased t‐Hcy in HD is consistent with impaired transsulfuration due to mutant HTT‐mediated dysregulation of SP1, ATF4, CBS, and CSE, leading to homocysteine accumulation and oxidative stress.^10^, ^12^, ^13^, ^14^, ^27^

Marked interindividual variability in t‐Hcy suggests the presence of additional modifying factors. Two profiles emerged: HHcy associated with vitamin B deficiency (20.8%) and isolated HHcy (29.9%). While nutritional deficiency may explain the former, the mechanisms underlying isolated HHcy remain unclear. One possible hypothesis is that genetic factors, particularly variants affecting 1‐CM, could contribute to altered homocysteine metabolism in a subset of HD patients. Further investigations are currently ongoing in our cohort to explore the potential role of 1‐CM‐related genetic variants in HD. Although no prior study has examined these variants in HD, earlier reports on *MTHFR‐1298CC* polymorphism yielded conflicting results, underscoring the need for further investigation.^15^, ^28^

Clinically, HHcy was associated with faster motor progression and correlated with UHDRS‐TMS/MD (*P < 0.001*), independent of age and CAG repeats, suggesting a possible relationship between altered HHcy and disease severity. This aligns with prior work suggesting t‐Hcy as a biomarker of ongoing neurodegeneration in HD, supported by the positive correlation between t‐Hcy and UHDRS scores reported by Andrich et al. in mildly affected untreated patients.^26^

Importantly, similar associations have been reported in other neurodegenerative disorders, notably PD, supporting a broader cross‐disease relevance of HHcy. Bakeberg et al.^7^ reported a positive correlation between t‐Hcy and Movement Disorder Society Unified Parkinson's Disease Rating Scale‐Part III (MDS‐UPDRS‐III Motor) scores in men, and a negative correlation between t‐Hcy and ACE‐R cognitive scores (Addenbrooke's Cognitive Examination‐Revised) in women with PD and HHcy, compared with PD controls without elevated t‐Hcy. Moreover, in an early‐stage PD cohort,^8^ low vitamin B12 levels at diagnosis predicted greater motor deterioration, while elevated t‐Hcy predicted greater cognitive deterioration over 2 years.

Together, these converging findings across HD and PD strengthen the hypothesis that HHcy is not disease‐specific but may represent a shared metabolic mechanism contributing to neurodegeneration and clinical progression in multiple neurodegenerative diseases.

HHcy was particularly prevalent in premanifest and early symptomatic stages, consistent with models proposing early neurodevelopmental vulnerability followed by accelerated neurodegeneration.^29^, ^30^, ^31^ HHcy may represent a metabolic marker associated with neurodegenerative processes in HD. By inducing oxidative stress, HHcy may promote somatic CAG expansion through impaired DNA repair,^32^ facilitating attainment of the pathogenic repeat threshold—beyond which cells become vulnerable— and triggering neurodegeneration.^33^ This mechanism may also explain the lower prevalence of HHcy in patients with longer disease duration, as faster progression could lead to earlier advanced disease, institutionalization, or death, reducing their likelihood of follow‐up.

We also observed an association between HHcy and behavioral‐dysexecutive onset, consistent with data from AD linking elevated t‐Hcy to behavioral symptoms.^34^, ^35^ These results support our finding that behavioral symptoms are more frequent as an initial sign in HD patients with HHcy.

These findings suggest that homocysteine metabolism may deserve further investigation in HD, particularly in relation to nutritional status, behavioral symptoms, and disease progression. Whether monitoring or correcting HHcy could have clinical benefit remains to be determined in prospective longitudinal studies. Therapeutic strategies targeting the transsulfuration pathway—through cysteine supplementation^12^ or CSE induction^36^—have shown promising results in experimental HD models and may justify further translational investigation.

Pathophysiologically, HHcy is a risk factor for several neurodegenerative diseases^4^, ^37^ and has harmful effects, such as N‐homocysteinylation.^38^, ^39^, ^40^ Over 80% of circulating Hcy is protein‐bound. Hcy binds proteins via reversible S‐homocysteinylation at normal t‐Hcy or irreversible N‐homocysteinylation in the case of HHcy, which leads to protein aggregation and dysfunction.^41^, ^42^, ^43^ Bossenmeyer et al.^37^ reported increased N‐homocysteinylation of tau and other microtubule‐associated proteins (MAPs) in the brain of those affected by AD, disrupting their protein interactions and aggravating their aggregation. We hypothesize that HHcy in HD may similarly promote protein aggregation via N‐homocysteinylation, contributing to age‐related neurodegeneration.

The strengths of this study include its prospective design, sample size, and inclusion of premanifest patients. A limitation of our study is that the motor progression rate was calculated using the patient‐reported age at onset. However, this information was collected independently of participants' clinical or biological status, thereby minimizing potential bias. Another concern that could be raised is that the UHDRS‐TMS score does not remain linear beyond the early stages of HD. Indeed, after the first few years of motor progression, score saturation reduces the linearity of UHDRS‐TMS changes.^18^, ^19^, ^20^, ^21^, ^22^ To address this limitation, we conducted an additional analysis restricted to participants with a motor disease duration of less than 5 years, in order to minimize the potential bias introduced by ceiling effects of the UHDRS‐TMS and inaccuracies in the reported age at onset. Another significant limitation of our study is the absence of cognitive and functional assessments, which are now recommended in HD clinical research.^44^

Future longitudinal studies should integrate composite clinical outcomes with repeated biomarker assessments to validate HHcy as a modifiable contributor to HD progression and to evaluate HHcy‐lowering interventions.

## Conclusions

This study identified significantly higher plasma homocysteine levels and an increased prevalence of hyperhomocysteinemia in both symptomatic and premanifest HD patients compared with controls. Elevated homocysteine levels were associated with markers of disease severity, supporting a potential link between altered one‐carbon metabolism and HD clinical expression. However, given the observational nature of the study, these findings should be interpreted cautiously and do not establish a causal relationship. Further longitudinal studies integrating metabolic, nutritional, and genetic data are required to better understand the role of homocysteine metabolism in HD pathophysiology and progression.

## Author Roles

(1) Research Project: A. Concept, B. Design, C. Material Preparation, D. Data Analysis; (2) Statistical Analysis: A. Design, B. Execution, C. Review and Critique; (3) Manuscript Preparation: A. Writing of the First Draft, B. Review and Critique, C. Editing of the Final Version.S.P.: 1A, 1B, 1C, 1D, 2B, 3A, 3C.

E.P.: 1A, 1B,

C.B.: 3B.

L.H.: 3B.

S.F.: 3B.

M.B.: 3B.

V.R.: 3B.

M.W.: 3B.

N.S.: 3B.

L.L.: 3B.

A.O.: 3B.

A.J.: 3B.

I.R.: 3B.

R.‐M.G.‐R.: 1A, 1B, 2B, 3B.

J.L.G.: 1A, 1B, 3B.

C.B.‐P.: 1A, 1B, 3B.

M.R.: 1A, 1B, 3B.

## Financial Disclosures and Conflicts of Interest

Author disclosures are available in the Supporting Information.

## Supporting information


**Figure S1.** Metabolism of homocysteine. Homocysteine is formed following the use of methionine as a methyl‐group donor. Homocysteine is disposed of through remethylation to methionine (using methionine synthase) or by transsulfuration to cystathionine (using cystathionine β‐synthase (CBS) and cystathionine γ‐lyase (CSE)). Methionine synthase requires the folic acid derivative 5‐methyl tetrahydrofolate (THF) as a substrate. The rate sensitive enzyme in the formation of 5‐methyl THF is 5,10‐methylenetetrahydrofolate reductase (MTHFR). Methionine synthase requires vitamin B12 as a cofactor; CBS and CSE require vitamin B6 as a cofactor.

**Table S1.** Characteristics of the controls (N=87) included in the study.
**Table S2:** One‐carbon metabolism disorders of the symptomatic Huntington's disease population.
**Table S3:** Characteristics of the premanifest Huntington's disease population.
**Table S4:** One‐carbon metabolism disorders of the premanifest Huntington's disease population.

**Disclosure.** Supporting Information.

## Data Availability

The data that support the findings of this study are available on request from the corresponding author. The data are not publicly available due to privacy or ethical restrictions.
