# Supplementary material for: Hyperhomocysteinemia and Vitamin B Deficiency as Potential Aggravating Factors in Huntington's Disease: A Prospective Monocentric Study
Source: Mov Disord. 2026 Jul 1;41(10):2712–22. doi: 10.1002/mds.70413 (PMC13686121; doi:10.1002/mds.70413)
Supplement: Supplementary file 2 [file MDS-41-2712-s002.docx]

**Supplemental data : Tables**

| Table 1: Characteristics of the 87 controls included in the study | | |
| --- | --- | --- |
| Demographic data | n |  |
| Age (years), mean ± SD |  | 50.2 ± 12.2 |
| Sex (M/F), n |  | 51/36 |
| Folates, serum level, > 7 nmol/L, mean ± SD | 84/87 | 20.8 ± 7.4 |
| Vitamin B-12 level, > 150 pmol/L, mean ± SD | 87/87 | 338.8 ± 95.9 |
| Homocysteine level, < 15 μmol/L, mean ± SD | 87/87 | 12.8 ± 2.7 |
| Methylmalonic acid, μmol/L, mean ± SD | 63/87 | 0.16 ± 0.06 |
| Anormal Methylmalonic acid, %(n) |  | 0 |
| Glomerular filtration rate, mL/min, mean ± SD | 67/87 | 92 ± 7.4 |
| No renal failure, >89 mL/min, %(n) |  | 88.1 (59) |
| Mild renal failure, 60-89 mL/min, %(n) |  | 11.9 (8) |
| Moderate renal failure, 30-59 mL/min, %(n) |  | 0 |
| ASAT, U/L, mean ± SD | 69/87 | 27.1 ± 7.6 |
| ALAT, U/L, mean ± SD | 69/87 | 33.7 ± 11.5 |
| Biological abnormalities |  | % (n) |
| Isolated hyperhomocysteinemia |  | 17.2 (15) |
| Isolated vitamin B-9 deficiency |  | 0 |
| Isolated vitamin B-12 deficiency |  | 0 |
| Hyperhomocysteinemia and vitamin B-12 deficiency |  | 0 |
| Hyperhomocysteinemia and vitamin B-6 deficiency |  | 0 |
| Hyperhomocysteinemia and vitamin B-12 and B-6 deficiencies |  | 0 |
| Hyperhomocysteinemia and vitamin B-12 and B-9 deficiencies |  | 0 |
| Abbreviations: SD = Standard deviation, UHDRS = Unified Huntington’s Disease Rating Scale. | | |

| **Table 2: One-carbon metabolism disorders of symptomatic HD population** |  |
| --- | --- |
|  | n=62 |
|  | % (n) |
| Hyperhomocysteinemia | 48.4 (30) |
| Isolated hyperhomocysteinemia | 24.2 (15) |
| Isolated vitamin B-6 deficiency | 11.3 (7) |
| Isolated folate deficiency | 0 |
| Isolated vitamin B-12 deficiency | 3.2 (2) |
| Hyperhomocysteinemia and vitamin B-12 deficiency | 1.6 (1) |
| Hyperhomocysteinemia and vitamin B-6 deficiency | 19.4 (12) |
| Hyperhomocysteinemia and vitamin B-12 and B-6 deficiencies | 1.6 (1) |
| Hyperhomocysteinemia and vitamin B-12 and folate deficiencies | 1.6 (1) |
| Abbreviations: HD = Huntington’s disease. | |

| Table 3: Characteristics of premanifest HD population |  |  |  |
| --- | --- | --- | --- |
|  | Patients without HHcy or vitamin B deficiency n=6 | Patients with HHcy or vitamin B deficiency n=9 | P Value |
|  | Mean ± SD | Mean ± SD |  |
| CAG repeat, longer allele | 43.3 ± 5.1 | 42.6 ± 2.27 | 0.6 |
| CAG repeat, shorter allele | 19.5 ± 2.62 | 18 ± 2.9 | 0.5 |
| Age, years | 42.8 ± 11.9 | 36 ± 6 | 0.31 |
| Vitamin B-6 level, nmol/L | 38 ± 19 | 46 ± 47 | 0.95 |
| RBC folate level, nmol/L | 338 ± 68 | 330 ± 115 | 0.39 |
| Vitamin B-12 level, pmol/L | 864 ± 166 | 985 ± 159 | 0.46 |
| Homocysteine level, μmol/L | 11.6 ± 2.15 | 23.1 ± 9.6 | <0.001* |
| *Considered significant |  |  |  |
| Abbreviations: HD = Huntington’s disease, OMD = One-carbon metabolism disorder, SD = Standard deviation. | | | |

| **Table 4: One-carbon metabolism disorders of premanifest HD population** |  |
| --- | --- |
|  | n=15 |
|  | %(n) |
| Hyperhomocysteinemia | 60 (9) |
| Isolated hyperhomocysteinemia | 53.3 (8) |
| Hyperhomocysteinemia and vitamin B-6 deficiency | 6.7 (1) |
| Abbreviations: HD = Huntington’s disease | |
